# Remarks on the Preceding Case

**Published:** 1784

**Authors:** 


*Remarks on the preceding caſe, by Mr.* R. Mynors.

IN a little treatiſe, which I lately publiſhed, under the title of 'Practical Thoughts on Amputation, &c.' after deſcribing the operative as well as curative proceſs of the particular mode of amputating therein recommended, and faithfully relating the ſucceſs which had attended it, I obſerved, that an equal degree of ſucceſs had attended a ſimilar method of treatment in caſes of wounds, formed by the extirpation of various ſpecies of diſeaſed tumors as well as of large accidental recent wounds of different kinds.

The

The caſe of a fractured ſkull, juſt now related, may ſerve as a farther illuſtration of the doctrine laid down in the work in queſtion.

Having long lamented the great deficiencies which the practice of ſurgery, in caſes of this ſort, labours under, and having my mind ſtrongly impreſſed with the idea of uniting divided parts by the firſt intention, after all operations in ſurgery that could poſſibly admit of it, I was induced to put in practice the above-mentioned treatment, hoping that it would be found capable of obviating a great many evils, which are too generally obſerved to follow the operation of the trephine, when conducted upon the plan hitherto commonly adopted; and I am happy in being able to obſerve, that the ſucceſs of this new mode of treatment in the preceding caſe has eſtabliſhed, as clearly and ſatisfactorily as a ſingle caſe can, the propriety of my opinion on this ſubject.

But if it ſhould be aſked, whether from the very expeditious cure, and the remarkably mild ſymptoms after the operation, which happened in this caſe, the ſame treatment indiſcriminately uſed in all ſimilar caſes, may be expected to be equally ſucceſsful, I anſwer in the negative; being too well convinced, that the brain brain and its meninges, from various external causes, may frequently suffer such great violence, as to render vain our best and most improved attempts to save the patient.

Nevertheless, in all those cases, where reasonable hopes of success may be entertained, I cannot be too solicitous in recommending the aforesaid practice, and in desiring the surgeon to consider the very different effects which must be produced in the constitution by such opposite methods as the one hitherto generally followed, and that which has been now described; the former exposing a large surface of dura mater, &c. to a *suppurative inflammation*, and all its consequences, while the latter solicits nature to employ her friendly process, the *adhesive inflammation*, and close up the wound with mildness and dispatch.

